# Auditory evoked potential electroencephalography-biometric dataset

**DOI:** 10.1016/j.dib.2024.111065

**Published:** 2024-10-28

**Authors:** Nibras Abo Alzahab, Angelo Di Iorio, Luca Apollonio, Muaaz Alshalak, Alessandro Gravina, Luca Antognoli, Marco Baldi, Lorenzo Scalise, Bilal Alchalabi

**Affiliations:** a Department of Information Engineering (DII). Marche Polytechnic University, Ancona Italy; b Department of Industrial Engineering and Mathematical Sciences (DIISM). Marche Polytechnic University, Ancona Italy; c Department of Electrical Engineering, École de Technologie Supérieure, Université du Québec, Canada

**Keywords:** Authentication, Auditory stimuli, Resting state, Bone-conducting headphones, EEG, Brain–computer interface (BCI)

## Abstract

This work aims to assess the use of electroencephalographic (EEG) signals as a means of biometric authentication. More than 240 recordings, each lasting 2 min, were gathered from 20 subjects involved in the data collection. Data include the results of experiments performed both in a resting state and in the presence of auditory stimuli. The resting-state EEG signals were acquired with both open and closed eyes. The auditory stimuli EEG signals consist of six experiments divided into two scenarios. The first scenario considers in-ear stimuli, while the second scenario considers bone-conducting stimuli. For each of the two scenarios, experiments include a native language song, a non-native language song and some neutral music.

This data could be used to develop biometric systems for authentication or identification. Additionally, they could be used to study the effect of auditory stimuli such as music on EEG activity and to compare it with the resting state condition.

Specifications Table

**Table:** 

Subject	Biomedical Engineering
Specific subject area	Brain-Computer Interface (BCI), Biometrics
Type of data	Time-Series
How the data were acquired	OpenBCI Ganglion Board, 200 Hz sampling rate, four channels: T7, F8, Cz, and P4. Gold Cup Electrodes. Ten20 Conductive Paste. Software: OpenBCI GUI · v5.0.3
Data format	Raw: EEG time series Segmented: EEG time series Filtered: EEG time series
Description of data collection	The data was acquired using a Ganglion Board from an OpenBCI manufacturer. The board samples the brain's electric activity, called electroencephalography (EEG), from the surface of the head. This signal is sampled at a 200 Hz sampling frequency with 32-bit accuracy for an analog-to-digital converter.
Data source location	*• Institution: Marche Polytechnic University* *• City/Town/Region: Ancona/Ancona/ Marche* *• Country: Italy* *• Latitude and longitude (and GPS coordinates, if possible) for collected samples/data: 43.587245, 13.515500*
Data accessibility	Repository name: Physionet DOI: 10.13026/ps31-fc50
Related research article	Abo Alzahab, N., Di Iorio, A., Baldi, M., & Scalise, L. (2022, October). Effect of auditory stimuli on electroencephalography-based authentication. In *2022 IEEE International Conference on Metrology for Extended Reality, Artificial Intelligence and Neural Engineering (MetroXRAINE)* (pp. 388–392). IEEE. 10.1109/MetroXRAINE54828.2022.9967652

## 1 Value of the Data

• The data provides the ability to make several types of comparisons and assessments, concerning:
  ○ Auditory Stimuli vs Resting State
  ○ Auditory stimuli conduction method (in-ear or bone conducting)
  ○ Language of auditory stimuli
• Several groups can benefit from this data:
  ○ Researchers in the field of
    ■ EEG signal processing and analysis
    ■ Biometric authentication/identification.
  ○ Newcomers to the field of BCI. This is due to a single dataset experiment's simplicity and low computation complexity.
• The data could be reused to
  ○ Understand the difference between auditory stimulation and the EEG signal.
  ○ Develop an EEG-based biometric system.

## 2 Background

The development of biometric systems is rapidly advancing, and the use of EEG, or brainwave signals, is gaining interest in robust authentication applications. This novel dataset explores how EEG signals respond to auditory stimuli, such as sounds delivered through traditional earphones and innovative bone-conducting headphones. Bone-conducting headphones are included to examine if they affect the brainʼs response differently, potentially improving the accuracy of EEG-based authentication systems. The experiment was set up to record brainwaves from specific areas of the brain using a simplified setup of just four electrodes. This approach balances detailed brain activity capture with a practical and cost-effective setup. Participants listened to various sounds, including songs in both their native and non-native languages, as well as neutral music. This variety was chosen to stimulate different brain responses, enhancing the system's ability to authenticate individuals under various conditions. This dataset offers new insights into the use of bone-conducting headphones for EEG and simplifies the recording technique. The results could lead to more secure and practical biometric authentication systems, broadening the applications of EEG in everyday technology.

## 3 Data Description

The Dataset is stored in .csv and WFDB formats. The names of the recordings are formatted as follows: s*XX*_ex*XX*_s*XX*: s(*Subject Number*)_ex(*Experiment Number*)_s(*Session Number. Just for ex01 and ex02*).

Example: s03_ex02_s01: Subject 03 Experiment 02 Session.

The Data is provided in three folders:

1. ***Raw Data:*** Each record contains the original dataset without segmenting or filtering.
2. ***Segmented Data:*** Each record contains two minutes of EEG signals with minimal noise. Data segmentation was performed manually, and the initial and final positions of the trimming points are listed in the appendix Excel file: data_trim.csv.
3. ***Filtered Data:*** Each record contains a filtered two-minute version of the EEG signals; the segmented data after applying a 50 Hz notch filter with a quality factor of 30 and a 1st order 1–40 Hz Butterworth filter.
4. ***WFDB_Files:*** Includes the same data in the three folders above but with Waveform Database format. This format contains *MIT Format* and *European Data Format (EDF)*.

**Fig. 1:**
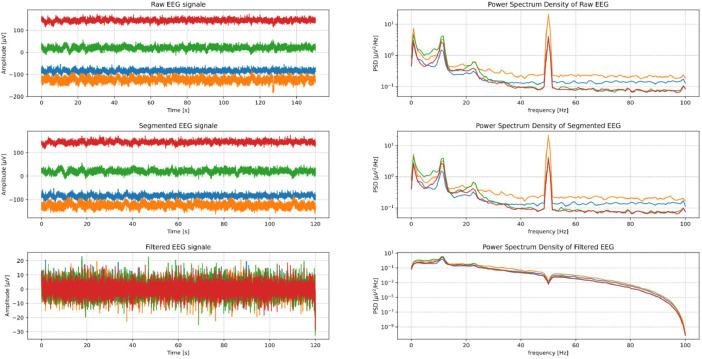
EEG signals of the three subsets.

The three versions of the signals are shown in Fig. 1. as an example of the data.

Appendices files:

• *Data_trim.csv:* The start and end points where the Segmented Data was trimmed from the Raw Data. The trimming points are annotated in seconds and samples.
• RECORDS: A list of directories to access the data stored in WFDB_Files.
• *Subjects.csv:* Contains the questionnaire's answers and the metadata of the subjects.
• *Songs.csv:* Contains links for the songs used in the recordings.

*Note 1:* Each filtered and segmented data file contains five columns: Sample index and four EEG channels.

*Note 2:* Each file in the raw data contains additional columns that can be discarded, but it was kept providing raw recording files. The first five columns are important columns: Sample index and four EEG channels.

*Note 3*: For the comments in *Subjects.csv:* file. Each subject was asked whether he was fluent in the non-native language.

## 4 Experimental Design, Materials and Methods

Brain-computer interface (BCI) systems are emerging and are being used beyond the medical field. However, the main problem of medical BCI systems is generalizability [1], which is the primary motivation for developing BCI systems for identity verification [2]. This dataset was created to investigate these biometric applications of EEG signals. Some results were presented at the 2022 IEEE International Conference on Metrology for eXtended Reality, Artificial Intelligence and Neural Engineering [3].

### 4.1 Determination of eligibility

The subjects filled out a questionnaire that contained the subjects’ information. Additionally, the subjects read and agreed to a detailed informed consent.

### 4.2 Preparation and installation of equipment

The experiments begin with the installation of the necessary equipment. Four gold-cup electrodes with Ten20 Conductive Paste have been used on the scalp. According to the 10/10 international EEG system [4], the electrodes were placed on the T7, F8, Cz, and P4 positions, which were determined based on works [[5], [6], [7]]. In addition, two electrodes were placed in the left and right ears as a reference and ground electrode, respectively. There are several motivations for selecting those locations. They are as follows:

1. The limit of choosing only four channels was due to available hardware, the OpenBCI Ganglion Board (Ganglion Getting Started Guide: OpenBCI Documentation, 2021) [8].
2. To find the best channel locations, insights from the literature were considered. Fig. 2 shows our channels compared to [6,7].

**Fig. 2:**
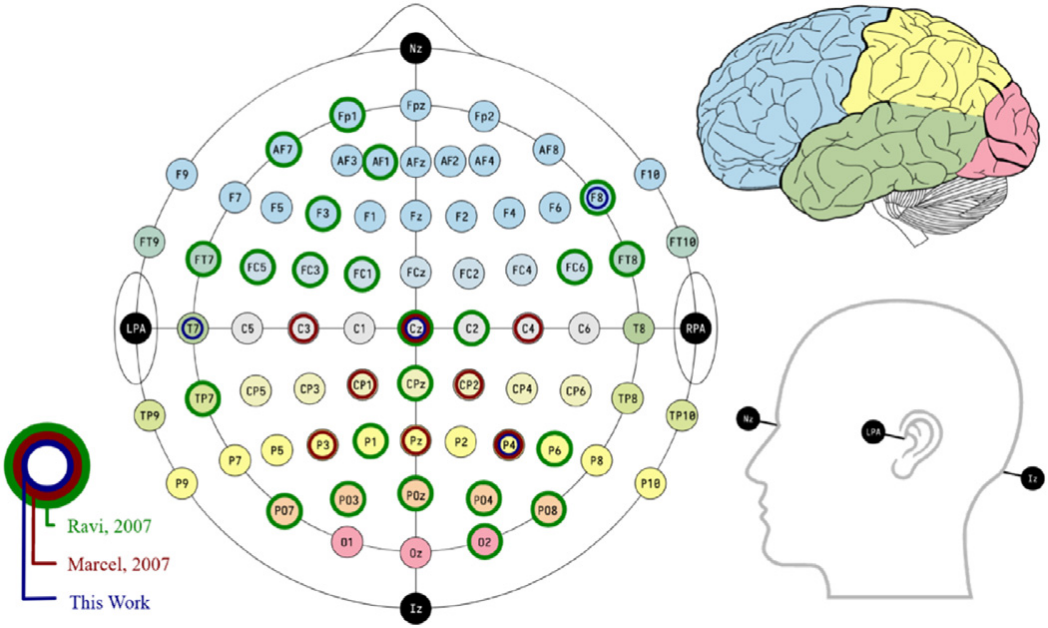
Channels locations.
3. Assortment in the parts of the cortex (Temporal (T7), Frontal (F8), Central (Cz), and Parietal (P4)) covered.

A video was created to show and describe the recording process. The video was uploaded to YouTube and can be found by searching [9]. After installing all the electrodes, a simple calibration was performed to ensure everything worked properly. Calibration included testing connectivity.

### 4.3 Testing and data recording

**Fig. 3:**
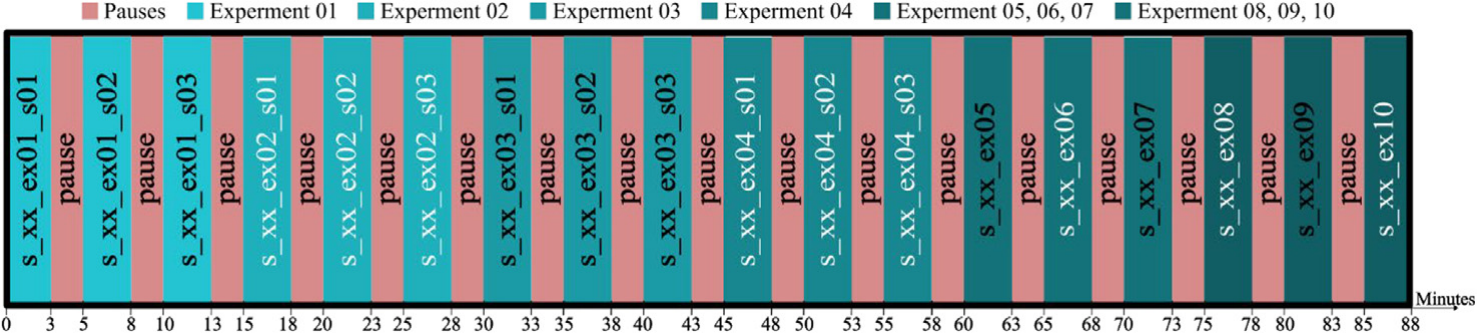
Experimental protocol of the EEG recording sessions.

The subject was asked to sit down and relax on a comfortable chair. The recording was performed on a single day per subject, and the tasks were in the same order. Between each experiment and the next one, subjects were provided with 2-min pauses, allowing for both a brief rest and the opportunity to review and manually assess the quality of the recorded data. Notably, experiments 1 through 4 were repeated across three sessions, all conducted on the same day, to generate additional recordings. This repetition ensures data reliability and depth for further analysis. Fig. 3 illustrates the overall protocol for the EEG recordings. The EEG data were recorded for 3 min, while the best 2 min (with no movement artefacts) were segmented and filtered as a final dataset.

The order of the experiments is the same. This can achieve consistency across subjects and minimize confounding variables. The recording involves the acquisition of the electroencephalographic signal as follows:

1. Two minutes of resting state, eyes open for three sessions.
2. Two minutes of resting state, eyes closed for three sessions.
3. *Non-Related experiment (Not provided in the dataset).*
  Two minutes of resting state, eyes open for three sessions using a noise isolation headset.
4. *Non-Related experiment (Not provided in the dataset).*
  Two minutes of resting state, eyes open for three sessions using a noise isolation headset.
5. Two minutes of listening to a song in their native language using in-ear headphones.
6. Two minutes of listening to a song in a non-native language using in-ear headphones.
7. Two minutes of listening to neutral music using in-ear headphones.
8. Two minutes of listening to a song in their native language using bone-conducting headphones.
9. Two minutes of listening to a song in a non-native language using bone-conducting headphones.
10. Two minutes of listening to neutral music using bone-conducting headphones.

*Note 1:* If the person is Italian: The Arabic song was non-native.

*Note 2:* If the person is not Italian, The Italian song was used as the non-native song.

*Note 3:* Neutral music is a musical genre that emphasises tranquility, relaxation, and peaceful soundscapes. It is typically composed of instrumental music (music with no or wordless vocals), and it may involve acoustic instruments, electric synthesisers, and even recorded nature sounds.

*Note 4: Exact track details are available in a CSV file on the Physionet Repository:*https://physionet.org/content/auditory-eeg/1.0.0/Songs.csv.

### 4.4 Data acquisition and filtering

The data were recorded using *OpenBCI GUI v5.0.3* [9] into text files (.txt) for a duration superior to two minutes for each experiment. The files were given as input to a Python program that reads the text file immediately after the recording is finished and allows us to segment manually the best 2 min (with no movement artifacts) of the recording. Additionally, the GUI acquisition system applies two filters to the signal. Those filters are a 50 Hz notch filter with a quality factor 30 and a 1st order 1–40 Hz Butterworth filter. The code finally saves the three versions of the signals, raw, segmented, and filtered, into comma-separated values (.csv) format. Finally, another Python script was used to convert the .csv files into Waveform Database (wfdb) format using the *wfdb 1.0.6* library.

## Limitations

The dataset has a limitation due to using only four electrodes, constrained by the OpenBCI Ganglion board's hardware. While these electrodes are placed across different brain regions, the limited number might not capture the full complexity of brain responses to auditory stimuli, affecting the dataset's detailed analysis potential. Additionally, due to some organisational and administrative issues, we were not able to record the same subject on different days. Therefore, the sessions were recorded on the same day for each subject. However, future research will focus on more sessions in order to study the psycho-emotional effects of EEG-based biometrics.

## Ethics Statement

A detailed informed consent form was given to the participants to read and sign. The World Medical Association (WMA) Declaration of Helsinki was followed during the experiments. To safeguard the privacy of study participants, all their individually identifiable health information and identities are secured and cannot be accessed by unauthorised individuals. Note that Formal ethical approval was not obtained for this study since that ethical approval was not necessary.

## Credit Author Statement

**Nibras ABO ALZAHAB:** Conceptualization, Methodology, Software, Formal analysis, Investigation, Data Curation, Writing - Original Draft, Visualization. **Angelo DI IORIO:** Investigation, Formal analysis, Visualization, Data Curation, Writing - Original Draft. **Luca APOLLONIO**: Investigation, Data Curation. **Muaaz ALSHALAK**: Investigation, Data Curation. **Alessandro GRAVINA**: Investigation, Data Curation. **Luca ANTOGNOLI**: Resources. **Marco BALDI**: Methodology, Writing - Review & Editing, Supervision, Project administration, Funding acquisition. **Lorenzo SCALISE**: Methodology, Validation, Writing - Review & Editing, Supervision, Project administration. **Bilal ALCHALABI**: Conceptualization, Methodology, Validation, Writing - Review & Editing, Supervision.

## Data Availability

physionetAuditory evoked potential EEG-Biometric dataset (Original data).

## Appendix Supplementary materials

Image, application 1

## References

[1] Abo Alzahab N., Apollonio L., Di Iorio A., Alshalak M., Iarlori S., Ferracuti F., Porcaro C. (2021). Hybrid deep learning (hDL)-based brain-computer interface (BCI) systems: a systematic review. Brain Sci..

[2] Abo Alzahab N., Baldi M., Scalise L. (2021). 2021 IEEE International Symposium on Medical Measurements and Applications (MeMeA).

[3] Abo Alzahab N., Di Iorio A., Baldi M., Scalise L. (2022). 2022 IEEE International Conference on Metrology for Extended Reality, Artificial Intelligence and Neural Engineering (MetroXRAINE).

[4] Jurcak V., Tsuzuki D., Dan I. (2007). 10/20, 10/10, and 10/5 systems revisited: their validity as relative head-surface-based positioning systems. Neuroimage.

[5] Altahat S.H.Q. (2017).

[6] Ravi K.V.R., Palaniappan R. (2007). International Conference on Computational Intelligence and Multimedia Applications (ICCIMA 2007).

[7] Marcel S., Millán J.D.R. (2007). Person authentication using brainwaves (EEG) and maximum a posteriori model adaptation. IEEE Trans. Pattern Anal. Mach. Intell..

[8] “Ganglion Getting Started Guide: openBCI Documentation,” 2021. https://docs.openbci.com/GettingStarted/Boards/GanglionGS/ (accessed 27 September 2022).

[9] Abo Alzahab N., Di Iorio A., Apollonio L., Alshalak M., Gravina A., Antognoli L., Baldi M., Scalise L., Alchalabi B. (2021). Auditory evoked potential EEG-Biometric dataset (version 1.0.0). PhysioNet.

