# Supplementary material for: Auditory evoked potential electroencephalography-biometric dataset
Source: Data Brief. 2024 Oct 28;57:111065. doi: 10.1016/j.dib.2024.111065 (PMC11582748; doi:10.1016/j.dib.2024.111065)
Supplement: Supplementary file 1 [file mmc1.docx]

Appendix A: Informed consent

**Invitation to Participate in "iCap” & “Brain-Biometric" projects.**

This is an invitation to take part in a research study in the framework of "iCap & Brain-Biometric" projects. Before taking a decision, it is fundamental that you have all the information in order to participate in a more conscious and responsible way. We recommend you read the whole document and ask to the responsible for the project any question that may arise.

- **Duration**: Approximately 75 minutes.
- **Location**: Lab at Università Politecnica delle Marche.
- **Collected** **Data**:
  - General Information (Questionnaire).
  - Brain-waves (Electroencephalography (EEG)).
- **Tools**
  - OpenBCI Ganglion Board.
  - Gold Cup electrodes.
  - Ten20 Conductive Paste.
  - Software: OpenBCI GUI · v5.0.3
- **Expected** **results**:
  - ***iCap*** ***Project***: Creating a headset that minimizes interference in neuronal signals, and thus, improve the ability to understand the brain and conduct more accurate research in the future.
  - ***Brain-Biometric Project***: Developing Brain-Biometric system.

# Participation in the research project involves:

## Determination of eligibility

After explaining the recording protocol and signing it, the subject will have to fill a questionnaire to confirm that the eligibility criteria for participating in the study are met.

## Preparation and installation of equipment

The experiments start with the installation of the equipment needed. This includes 4 gold-cup electrodes with *Ten20* Conductive Paste on the scalp. Additional 2 electrodes will be placed in the left and right ears as a reference electrode and ground electrode. Once all electrodes have been installed, a simple calibration will be performed to ensure that everything is working properly. In the next step, the subject will be asked to wear a headset consisting of two rubber insulating layers containing an overlapping aluminum foil and mesh.

## Testing and data recording

The subject is asked to sit down and relax. The first session involves the acquisition of the electroencephalographic signal:

1. 5 minutes with iCap, eyes open.
2. 5 minutes without iCap, eyes open.
3. 5 minutes with iCap, eyes closed.
4. 5 minutes without iCap, eyes closed.

## Removing the equipment

Once the tests are completed, the headset and electrodes will be removed. Cleaning Material will be provided for removal of residual conductive gel from the head.

# Benefits, inconveniences and/or potential risks of participation

Participation in the study is voluntary and free of charge. For the participants, the collaboration does not involve any kind of risk.

# Withdrawal from this study

You have the right to withdraw your consent to participate in this study at any time, even without prior notice or specific reason.

# Return

You have the right to request information about the results, progress, and outcome of the research.

# Measures to protect anonymity

The data collected will be processed in such a way as to eliminate any references that might allow reviling the subject identity. The data and the results of the research will be published as a public dataset. The data might be used in further research.

I, the undersigned _____________________ residing at ________________, street _________________

DECLARES:

 I have read the above information sheet received, that I understand both the information contained therein, and the information provided orally by the iCap research project staff, and that I have had ample time and opportunity to ask questions, and obtain satisfactory answers from the staff;

 I understand that participation in the study is entirely voluntary and free, that I may withdraw from the study at any time, without explanation and without disadvantage or prejudice;

 I understand the nature, and activities involved in participating in the study, and the risks involved;

 I understand that participation in this study will not confer any direct or indirect financial advantage.

Accordingly, the undersigned

 Agrees  does not agree

to participate in the study, in the knowledge that such consent is freely given and may be revoked at any time without disadvantage or prejudice.

Place and Date: ________________, _________________

Signature: _________________________________

Signature of consent collector: _________________________________

**Recording Protocol and Data Analysis:**

The data is recorded using OpenBCI ganglion board with 4 channels in positions: T7, F8, Cz, and P4, according to [1,2,3]. The data is recorded in 4 sessions:

1. 5 minutes with iCap, eyes open.
2. 5 minutes without iCap, eyes open.
3. 5 minutes with iCap, eyes closed.
4. 5 minutes without iCap, eyes closed.

Additionally, 5 minutes of environment noise, before electrode placement is recorded.

The data acquisitioning software is *OpenBCI GUI · v5.0.3* using windows 64-bit version.

The data collecting and building the dataset is divided into 3 steps:

1. Testing step: 5-subjects. This step will provide a sense of data. To know whether the process needs modification or not.
2. Building step: 10-subjects. Ensure that the process is going well, and reliable data are being collected.
3. Increment step: 15-subjects: Increase the number of the dataset’s subject into a reliable number.

Data Processing:

1. Data cropping: Delete the first and last 30 seconds of the record, which mostly contain noise.
2. Filtering: apply a 5^th^ order 0.1 ~ 30 Hz Low-pass Butterworth filter.
3. Segmenting: segment the data into 5 seconds segments with 50% overlapping.

Data Analysis:

1. iCap Project:
   1. Calculate the noise to signal ratio for the 4 sessions.
   2. Compare the results between with and without iCap in case of eyes open.
   3. Compare the results between with and without iCap in case of eyes closed.
2. Brain-Biometric Project:
   1. Extract features.
   2. Apply classification algorithm.
   3. Performance evaluation.
   4. Compare results with dataset collected by an advanced acquisitioning system.

References:

[1] Altahat, S. H. Q. (2017). *Robust EEG Channel Set for Biometric Application* (Doctoral dissertation, University of Canberra).

[2] Ravi, K. V. R., & Palaniappan, R. (2007, December). A minimal channel set for individual identification with EEG biometric using genetic algorithm. In *International Conference on Computational Intelligence and Multimedia Applications (ICCIMA 2007)* (Vol. 2, pp. 328-332). IEEE.

[3] Marcel, S., & Millán, J. D. R. (2007). Person authentication using brainwaves (EEG) and maximum a posteriori model adaptation. *IEEE transactions on pattern analysis and machine intelligence*, *29*(4), 743-752.

Appendix B: Questionnaire

**Questionnaire**

1. Name: __________________________________________________________________
2. Surname: ________________________________________________________________
3. Age: ____________________________________________________________________
4. Gender: Male Female
5. Profession: _____________________________________________________________
6. Weight: ________________________________________________________________
7. Height: _________________________________________________________________
8. BMI: ___________________________________________________________________
9. Do you smoke or have you ever smoked: Yes No
10. Do you use alcoholic beverages: Usually Occasionally Never
11. Do you use long-term medications: Yes No
12. Mother Language: _____________
13. Order the of the recorded experiments (Eyes Closed, Eyes Open, In-Ear, Bone Conducting) according to your satisfaction:
    1. ______________
    2. ______________
    3. ______________
    4. ______________

Appendix C: Recording Sheet for calibration

**Recording Sheet for Subject: _______________**


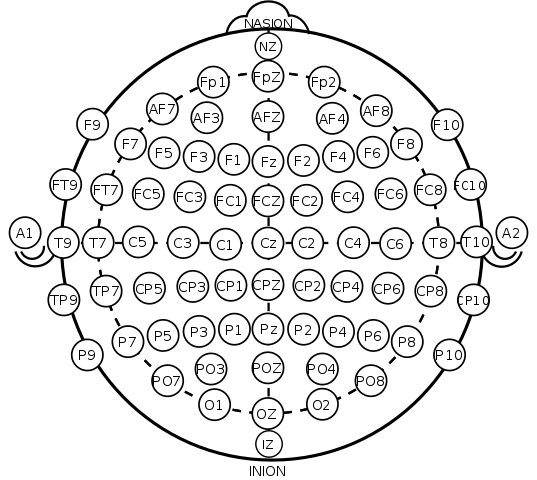


| E0 | T7 | White |
| --- | --- | --- |
| E1 | **F8** | **Orange** |
| E2 | **Cz** | **Gray** |
| E3 | **P4** | **Yellow** |

_____________________________________________________________________________________

| Impedance of | T7 [KΩ] | P8 [KΩ] | Cz [KΩ] | P4 [KΩ] | Ref [KΩ] |
| --- | --- | --- | --- | --- | --- |
| Experiment 01 |  |  |  |  |  |
| Experiment 02 |  |  |  |  |  |
| Experiment 03 |  |  |  |  |  |
| Experiment 04 |  |  |  |  |  |
| Experiment 05 |  |  |  |  |  |
| Experiment 06 |  |  |  |  |  |
| Experiment 07 |  |  |  |  |  |
| Experiment 08 |  |  |  |  |  |
| Experiment 09 |  |  |  |  |  |
| Experiment 10 |  |  |  |  |  |

Experiments:

1. Eyes Open – No iCAP:
   3min – 3 min 3 min
2. Eyes Close – No iCAP:
   3min – 3 min 3 min
3. Eyes Open – iCAP: 3min – 3 min 3 min
4. Eyes Close – iCAP: 3min – 3 min 3 min
5. Native (ear)
6. Non-native (ear)
7. Neutral (ear)
8. Native (ear)
9. Non-native (ear)
10. Neutral (ear)
